# Experiences of nurse educators regarding teaching and learning during the COVID-19 pandemic

**DOI:** 10.4102/hsag.v30i0.2882

**Published:** 2025-04-11

**Authors:** Kebuseditswe S. Gontse, Gaotswake P. Kovane, Isaac O. Mokgaola, Seepaneng S. Moloko-Phiri

**Affiliations:** 1 NuMIQ Research Focus Area, Faculty of Health Sciences, North-West University, Mahikeng, South Africa

**Keywords:** higher education institutions, COVID-19, experiences, nurse educator, nursing education institution

## Abstract

**Background:**

The abrupt start of the coronavirus disease 2019 (COVID-19) pandemic impacted educational systems globally, including nursing education institutions (NEIs). Teaching and learning in academic institutions were disturbed because of strict legislation measures, like lockdowns, that were implemented to curb the spread of COVID-19. These measures affected the quality of teaching and academic attainment.

**Aim:**

This study aimed to explore and describe the experiences of nurse educators regarding teaching and learning during the COVID-19 pandemic at a NEI in the North West province (NWP). The study was conducted at a selected NEI, which comprises two campuses that are 178.1 km apart.

**Method:**

A qualitative descriptive phenomenology approach was used. The nurse educators were purposively sampled, and 25 nurse educators participated in this study. Four focus group discussions (FGDs) were held to collect data through face-to-face semi-structured interviews. The data analysis followed Colaizzi’s seven-stage process.

**Results:**

Three themes emerged from data analysis: positive experiences and negative experiences regarding teaching and learning during the COVID-19 pandemic, and strategies for overcoming the challenges experienced by nurse educators during the COVID-19 pandemic.

**Conclusion:**

The study findings revealed that nurse educators experienced positive and negative challenges regarding teaching and learning during the COVID-19 pandemic. Furthermore, there is a need for NEIs to employ teaching and learning innovations, including the use of technology, to be able to divert to online teaching and learning during future pandemics where face-to-face teaching is halted.

**Contribution:**

The study findings may provide valuable insights for policymakers in higher education institutions (HEIs) to develop policies that incorporate innovative teaching methods. These methods will be relevant for future pandemics that may disrupt traditional face-to-face teaching and learning. As an enhancement of existing teaching practices, innovative approaches have proven to be effective and contribute to more inclusive education. By adopting these methods, HEIs can better support the achievement of Sustainable Development Goal 4, which aims to ensure quality and equitable education for all.

## Introduction

The coronavirus disease 2019 (COVID-19) pandemic during 2019–2021 was one of the deadliest diseases the world has ever experienced. The COVID-19 pandemic brought the world to a standstill, disrupting normal working conditions (Makhado et al. 2022:2). The abrupt outbreak of COVID-19 had an impact on educational systems globally, and nursing education institutions (NEIs) were not excluded (Molefe & Mabunda 2022:1; Wallace et al. 2021:612). According to a study conducted in Namibia, COVID-19 distracted teaching and learning in higher education institutions (HEIs) (Shindjabuluka, Ashipala & Likando 2022:1). The spread of COVID-19 infections was reportedly high in social gatherings, including HEIs (Whaley et al. 2021:1090). Therefore, NEIs suspended face-to-face delivery of teaching and learning (Leaver, Stanley & Veenema 2022:82; Li et al. 2021:2).

Similarly, in Hong Kong, clinical placement was also halted to reduce the rate of COVID-19 infections (Cheung, Fong & Bressington 2021:116). However, some institutions continued with clinical teaching by allocating nursing students to clinical facilities (Leidl et al. 2020:3). Nursing education institutions had to devise measures to curb the spread of COVID-19 infections, such as using technology to continue rendering teaching and learning (Leaver et al. 2022:82; Shindjabuluka et al. 2022:1). Furthermore, the COVID-19 pandemic increased the demand for online teaching and learning (Gause, Mokgaola & Rakhudu 2022:1).

Nursing students and nurse educators experienced challenges regarding teaching and learning because of the COVID-19 pandemic (Cheung et al. 2021:116). Challenges such as the rapid transition to online teaching and learning technology without time for preparation and orientation were reported (Leaver et al. 2022:82). Furthermore, nurse educators were challenged to restructure the course content’s delivery and evaluation (Leaver et al. 2022:82). In Iran, nurse educators also experienced difficulties regarding home-based working, whereby there used to be noise in the background, which affected teaching (Farsi et al. 2021:9). Furthermore, nurse educators experienced challenges in creating teaching slides, which were, sometimes, too large to send via email (Farsi et al. 2021:9).

Additionally, owing to the sudden and abrupt implementations of strict no-movement regulations because of the COVID-19 outbreak, specific formats such as Microsoft PowerPoint presentations were not provided face to face. Online education could only meet some of the needs because of challenges with internet access and connectivity. Some students lacked internet access, while others experienced poor connectivity and some of the slides could not be opened (Farsi et al. 2021:9). Similarly, in another study, it has been reported that nurse educators had negative attitudes towards online teaching and learning because of a lack of internet access and poor digital skills (Kunaviktikul et al. 2022:2). According to a study conducted in South Africa, clinical teaching and learning were challenging to achieve because of restrictions brought by COVID-19, such as no more than 50 people at a gathering and social distancing rule (Makhado et al. 2022:9). The authors further assert that practical skills and simulation sessions were challenging to simulate online as these skills require interaction and psychomotor skills. In addition, it has been reported that most students in Pakistan claimed that e-learning was ineffective in attaining their clinical skills (Abbasi et al. 2020:4).

Apart from teaching and learning disruptions that were brought about by the COVID-19 pandemic, psychological challenges were experienced worldwide. For example, in Iran, some nurse educators had increased worries, fears and anxiety about being infected with COVID-19 and probably transmitting the disease to their children and family (Kaveh et al. 2022:6). The authors further stated that nurse educators experienced psychological stressors from numerous governmental directives. Similarly, another qualitative study documented that little information about the disease and the escalating number of infections and deaths added more to the confusion and anxiety levels of nurse educators (Farsi et al. 2021:7).

At the selected NEI in the North West province (NWP), the accredited teaching mode by the South African Nursing Council (SANC) is contact teaching and learning. However, the teaching mode was rapidly changed to online teaching, resulting in numerous challenges for nurse educators and students as they were not conversant with the method. South African government also incorporated lockdown measures that were implemented in five levels according to the needs of the country, to save lives and curb the spread of COVID-19 disease (Staunton, Swanepoel & Labuschaigne 2020:2). As the alert levels of lockdown changed, NEIs had to gradually re-introduce contact teaching and learning. Therefore, this study aims to explore and describe the experiences of nurse educators regarding teaching and learning during the COVID-19 pandemic at selected NEIs in the NWP.

## Methods

A qualitative descriptive phenomenology approach was used to explore and describe the lived experiences of nurse educators regarding teaching and learning during the COVID-19 pandemic. Descriptive phenomenology is concerned with defining lived experiences, and it focuses on how people interpret and make sense of their experiences in the world (Polit & Beck 2017:667).

### Study setting

The study was conducted at a selected NEI, which comprises two campuses that are 178.1 km apart. The selected NEI is accredited for the new curriculum of R171 with effect from February 2021. The current student intake for the new curriculum of R171 is 40 per campus. One campus had an estimated number of 50 nurse educators. Nine of the nurse educators were in managerial posts, that is, eight heads of departments (HODs) and one campus head. Some had office spaces, while others were sharing in groups of five to seven and each nurse educator had either a desktop or a laptop. The other campus had an estimated number of 40 nurse educators. Ten of the nurse educators were in managerial posts and had their own offices. Thirty of the nurse educators shared an office space in a boardroom, and each one had either a desktop or a laptop. However, the nurse educators who were in the managerial posts were not involved in direct teaching and learning.

### Sampling technique

The nurse educators were purposively selected to participate in the study. The participants met inclusion criteria if they possessed either a diploma or degree in nursing with an additional qualification in nursing education and were registered under Section 31 of the *Nursing Act, 2005* (Act 33 of 2005). Potential participants who did not meet the inclusion criteria were excluded from participation. The sample size was determined by data saturation when no new information emerged. Therefore, 25 nurse educators participated in the study.

### Recruitment

After approval to conduct the study was obtained, the recruitment of participants adhered to the ethical principles of autonomy, beneficence and justice. The campus heads from the selected NEI were the gatekeepers to gain access to the participants. The participants who showed interest permitted the campus heads to give their email addresses to the researcher to conduct an information session via Google Meet, which the researcher facilitated. The signing of informed consent was facilitated by an independent person who further ensured that only the nurse educators who were teaching at the campuses of the selected NEI during the COVID-19 pandemic would be enrolled in the study. The participants consented to be audio recorded during interviews.

### Data collection

Data were collected using a semi-structured interview guide to conduct face-to-face focus group discussions (FGDs). The FGDs enabled the researcher to gain overall information about the lived experiences of nurse educators, and they further ensured alignment with research objectives and promoted consistency (Creswell & Poth 2016:61). The interview guide was developed in line with the aim of the study and was pilot tested by the authors. It comprised a list of four questions that facilitated the discussion. The authors created research questions by defining the research problem, reviewing existing literature, designing open-ended and participant-focused inquiries, ensuring feasibility and relevance, and aligning them with the research design to explore and describe the experiences of nurse educators regarding teaching and learning during the COVID-19 pandemic (Creswell & Poth 2016:107). The interviews were conducted by an independent person who holds a master’s degree and was still studying towards a Doctor of Philosophy in Nursing at the time of data collection.

Probing and prompts were employed by the interviewer to ensure that all aspects of the research were covered adequately (Polit & Beck 2017:720). The probing questions were built on previous responses that were vague, allowing participants to expand their views or narrow broad statements. Two FGD sessions were conducted at each campus of a selected NEI. Therefore, a total of four FGD sessions were held. The FGDs lasted approximately 45 min to 60 min and were audio recorded with the participant’s permission. The field notes were also taken. The duration of data collection was from October 2022 to January 2023. Data collection was prolonged because of the challenges of accessing the participants in a group form as nurse educators were having different commitments because the institution was running two nursing programmes concurrently. Interviews were conducted in English which is the official medium of communication at the selected NEI. Participants were encouraged to freely share their experiences; moreover, the researcher used several methods to build rapport which aided in gaining trust and obtaining more information about their experiences. The interviewer maintained eye contact during interviews, avoiding being judgemental and ensuring to pay respect to the participants at all times.

### Data analysis

The study followed an inductive approach. Colaizzi’s seven-stage process was used to analyse data. In stage one, the researcher transcribed the data from audio recorders immediately after the first interview was conducted, verbatim and read written data from the transcripts several times to get their meaning. In the second stage, significant statements were extracted. That is, only relevant information about the experiences of nurse educators regarding teaching and learning during the COVID-19 pandemic at the NEIs in the NWP was extracted from each description. The researcher organised data in a meaningful and systematic way. In the third stage, data were organised and broken into small chunks of text and segments (coding). In the fourth stage, the segments were divided into sentences (or paragraphs) and clustered into themes. In the fifth stage, themes relevant to the study were defined and supported by quotations and literature. In the sixth stage, the researcher reviewed and modified the themes identified in stage five, while in the seventh stage, the themes were refined and validated to determine what each topic was about. The researcher and co-coder independently created codes, themes and sub-themes.

### Trustworthiness

To ensure that the study is trustworthy, the researcher considered Guba and Lincoln’s criteria. The credibility, transferability, dependability and confirmability were considered to increase the believability of the data. Prolonged engagement with the participants was exercised. The researcher had extended engagement with the participants during the semi-structured FGDs to gain an in-depth understanding and rule out if there was no repeating information. The researcher carried out member checking by first analysing the raw data from the interviews and developing distinct descriptions or themes, which were shared with the participants to determine their accuracy. To increase dependability and confirmability, the research processes, including a detailed description of the research methods and transcripts, were given to an independent person for analysis and coding to ensure consistent findings.

### Ethical considerations

Ethical approval to conduct the study was granted by the North-West University Health Research Ethics Committee (ethics number: NWU-00081-22-A1). Authorisation to conduct the study was requested from the North West Provincial Department of Health (NWDoH) and the management of the two campuses of the selected NEI in the NWP. The researcher ensured that ethical principles were maintained in this study by ensuring that participants’ privacy and confidentiality were maintained and protected during the research. The interviews were held face to face and only the recruited participants were admitted. No research data were linked to the participants as their names were not used, but anonymous codes. A password was encrypted in all soft data, while hard copies were stored in a locked cupboard. Participants were permitted audio recording, and consent to participate in the study was obtained without coercion.

## Review findings

Twenty-five nurse educators participated in the study, consisting of 23 females and two males. Extracts from participants’ FGDs were used to support the descriptions of the themes. Findings for the study emerged with three themes and 10 sub-themes presented in Table 1.

**TABLE 1:** Summary of themes and sub-themes from the four FGDs with nurse educators.

Themes	Sub-themes
1. Positive experiences regarding teaching and learning during the COVID-19 pandemic.	1.1 Integration of technology in teaching and learning. 1.2 Introduction of hybrid teaching and learning. 1.3 Increased teamwork among staff members.
2. Negative experiences regarding teaching and learning during the COVID-19 pandemic.	2.1 Fear of contracting COVID-19 and dying. 2.2 Shortage of classrooms. 2.3 Abrupt changes in theoretical and clinical teaching and learning. 2.4 Difficulties in using technology for teaching and learning.
3. Strategies for overcoming the challenges experienced by nurse educators during the COVID-19 pandemic.	3.1 Compliance with all COVID-19 rules and regulations. 3.2 Vaccination of employees and students. 3.3 Special arrangement for extra classes and remedial assessments.

COVID-19, coronavirus disease 2019.

### Theme 1: Positive experiences regarding teaching and learning during the COVID-19 pandemic

Positive experiences regarding teaching and learning during the COVID-19 pandemic emerged as the first theme with the following sub-themes: integration of technology in teaching and learning, introduction of hybrid teaching and learning, and increased teamwork among staff members.

#### Sub-theme 1.1: Integration of technology in teaching and learning

The participants reported that even though they experienced different challenges during the COVID-19 pandemic, it also brought some positive experiences, such as improved digital skills.

One participant said:

‘We had to learn very fast to new technology, all of us now we really know how to operate Zoom meetings, we can effectively use other methods of uh, uh, technology which we were not well conversant with previously so those are some of the good things that COVID-19 brought to the nursing education and our college.’ (Participant A, Female, FGD 1)

Another participant added:

‘It also taught us to be technology-wise. Because really, when it came, the Zooms were not … they were there, but we were not really into technology.’ (Participant C, Female, FGD 3)

The outbreak of COVID-19 improved the digital skills of nurse educators and nursing students; they can communicate using different platforms such as Zoom, teams and others.

#### Sub-theme 1.2: Introduction of hybrid teaching and learning

Participants reported that the COVID-19 pandemic also exposed them to other modes of delivering teaching and learning. One participant said:

‘The other issue, COVID-19 has brought this new thing, our curriculum as approved by the South African Nursing Council, it was contact sessions, but automatically, we had to deviate from what we had previously agreed to. And later on, the good thing is that SANC allowed all institutions in South Africa to do both the contact and remote teaching, although initially the curriculum was not agreed to as such.’ (Participant A, Female, FGD 1)

Another participant added:

‘But the WhatsApp group, I must be honest, they helped a lot and, there were a few of them that we formed … that we could have like a … not like a teleconference, almost something like teams or something yeah … where we could interact verbally. The students and the lecturers could use technology and face-to-face at times.’ (Participant A, female, FGD 4)

Irrespective of the challenges brought by COVID-19, it improved the knowledge skills of nurse educators and nursing students regarding the use of technology.

#### Sub-theme 1.3: Increased teamwork among staff members

The participants reported that the COVID-19 pandemic improved teamwork and solidarity among the staff of the selected NEI.

One participant said:

‘What I wanted to say was that COVID-19 united us. Why am I saying it united us? Different stakeholders were responsible for setting duties. Let us take, for example, the security guy; his role, we know, was more of taking care of or protecting the college’s assets. During COVID-19, they even had to take the temperature of doctors and nurses at the hospital and the staff at the college, and if the temperature was not normal, there was no way they would allow us to go in. Meaning when you look at the role.’ (Participant A, Male, FGD 3)

Another participant added:

‘We were working as a team during COVID-19, like a family. There was no security, no cleaner, no nurse, we all wanted to work together to fight this disease, and here we are today. We have conquered it.’ (Participant C, Female, FGD 2)

Coronavirus disease 2019 improved the interpersonal skills of the employees of the institution. The statements confirm no discrimination in terms of categories and responsibilities.

### Theme 2: Negative experiences regarding teaching and learning during the COVID-19 pandemic

Negative experiences regarding teaching and learning during the COVID-19 pandemic emerged as the second theme. This theme was further divided into the following sub-themes: fear of contracting COVID-19 and dying, shortage of classrooms, abrupt changes in theoretical and clinical teaching and learning, and difficulties in using technology for teaching and learning.

#### Sub-theme 2.1: Fear of contracting COVID-19 and dying

The participants reported that they feared contracting COVID-19 while providing teaching and learning, as there was a high number of infections and an increased mortality rate related to COVID-19 infections. There was also increased absenteeism of educators and students because of infections.

One participant said:

‘Because of the uproar of COVID-19, there was fear, there was intense fear because of the number of infections, the number of deaths that were reported … really with that only, everybody was fearful.’ (Participant B, Female, FGD 2)

Another participant added:

‘… I would say I was afraid, fear of the infection because most people were dying, so we were at home and feared contracting COVID. Even when we had to work or go to the clinical facilities, we feared that we would be infected and die because of the coronavirus.’ (Participant H, Female, FGD 1)

Another participant echoed:

‘Some of the learners were put off, especially when we came to the quarantine when we were in class. They would be booked off because of COVID-19. If one is booked off, the whole class is not coming to work, they will be absent for ten days, and then on quarantine, we were having a real problem.’ (Participant B, Female, FGD 4)

Another participant commented:

‘We had a problem with placing the students in the clinical facilities because of the numbers, the number of students we could not place a large number of students at the same time because we were allocated numbers, the facility would tell us how many students we should place and also accompanying those students it was a problem because we feared infection from COVID-19.’ (Participant B, Female, FGD 1)

This indeed confirms that the outbreak of COVID-19 brought uncertainties. Some nurse educators and nursing students had increased worries, fears and anxiety about being infected with COVID-19 and probably transmitting the disease to their children and families.

#### Sub-theme 2.2: Shortage of classrooms

As the COVID-19 restrictions were progressively eased, contact sessions were allowed for teaching and learning; however, there were still restrictions to the capacity of classrooms. Participants reported that they needed more classrooms to accommodate social distancing. Therefore, students had to be divided into groups, and the institution had to hire venues to continue teaching and learning. They further reported that the hired venues could have been more convenient for teaching and learning. Repeating the same content leads to the exhaustion of educators, affecting the quality of teaching and learning.

One participant said:

‘When the students came back, we did not have enough classrooms because the ones we had were not big enough to can accommodate the social distancing that we were talking about 1.5 metres to 2 metres. One class that should accommodate 50 by then, it could only accommodate 25 or 30 students but still not stick to the social distancing principle of 1.5 metres.’ (Participant A, Male, FGD 3)

Another participant added:

‘For the college, it was very costly to hire venues and some of those venues were not user-friendly like the other venue where we were like they already said there were some problems, no effective ablution facilities, no water, no electricity and all that was affecting the teachers and the students, especially when there was no electricity.’ (Participant B, Female, FGD 1)

Another participant commented:

‘I remember we had around 54 students then, and we had to divide them into two groups. The first class was from 08:00 a.m to 12:00 a.m, and the other was from 12:30 a.m to around 16:30 p.m. So it was also very challenging because the same lecturer had to teach two groups the same content. The first group maybe will benefit because you are still flexible, teaching in whatever way you can and when the second group comes, you are a human being. You will be exhausted.’ (Participant C, Female, FGD 3)

The outbreak of COVID-19 also had financial implications because the venues were hired even though they did not have basic amenities such as electricity and an ablution block, among others. As a result, the selected NEI also had physical impacts on the nurse educators as the same content had to be repeated in different groups.

#### Sub-theme 2.3: Abrupt changes in theoretical and clinical teaching and learning

Participants reported that all the plenary actions, including the assessment programmes, had to be rescheduled. They further reported that some clinical skills induction tools had to be changed to abide by the COVID-19 rules. These changes also lead to the suspension of classes resulting in the extension of programmes.

One participant said:

‘Patients were no longer given mouth resuscitation, but now the tool was talking about mouth resuscitation, which was prepared before COVID, so some of the educators still insisted that students should do that, which was a hazard, and we had to include our Director of Nursing Education, and also we had to include all the South African Research Councils to have consensus as our examiners are centralised.’ (Participant A, Female, FGD 1)

Another participant added:

‘Indeed, it was a very critical period, and plans had to be rescheduled no matter what. We did our best to ensure that the clinical contact hours, as made by the South African Nursing Council, had to be covered. So, some students had to be allocated at night to push the clinical contact hours. However, occasionally they were contacted with the WhatsApp platform…you know they had to be reached somehow.’ (Participant F, Female, FGD 2)

Other participants added:

‘Since I had said that COVID-19 disrupted teaching and learning, it meant our students’ period of training was extended because of this disease or conditions.’ (Participant E, Female, FGD 2)

‘Due to the COVID-19 then and us working from home, the exam was then postponed for a whole six months…instead of them writing in May 2020, they wrote in November 2020.’ (Participant A, Female, FGD 4)

The statements indicate that the abrupt outbreak of the COVID-19 pandemic disrupted both theoretical and clinical teaching and learning. This resulted in rescheduling and finding alternative measures to meet the requirements as stipulated by the South African Nursing Council. However, the measures to continue teaching and learning needed to be revised, resulting in unavoidable course extensions.

#### Sub-theme 2.4: Difficulties in using technology for teaching and learning

During the level five lockdown, where contact sessions were not allowed, the NEI had to resort to online teaching and learning, which brought challenges. The challenges were either because of needing access to resources such as a computer and internet connectivity or data availability. Moreover, some needed better digital skills to access online platforms such as Zoom, Teams and others.

One participant said:

‘As far as educational issues are concerned, contact between students and lecturers was kept to a minimum so far that the only contact method was through WhatsApp. We could not zoom with students because most did not have cell phones that were compliant to access that service.’ (Participant D, Female, FGD 2)

Another participant added:

‘We experienced the same challenges. The thing was that most of the students were staying in rural areas, where there was no internet access. We struggled to reach a few using WhatsApp, but with others, we lost. We lost some of our students during the pandemic. We could not communicate with them, even just trying to call them. It was so difficult.’ (Participant G, Female, FGD 2)

Another participant commented:

‘Yeah, it was really difficult because we did not have resources. I did not have resources because I only had my laptop at home, and there was no data for me to communicate with the students.’ (Participant B, Male, FGD 3)

Another participant said:

‘We had frustration because of the use of technology, we did not know how to use Zoom, and it was the first time that we heard of it as an online teaching.’ (Participant H, Female, FGD 1)

One of the participants said:

‘As you know … uh what I can call it … facilities … students were given some cell phones … but some could not access the internet because of how far we are taking our students from … from the ruralness of the rural.’ (Participant B, Female, FGD 2)

One participant reported:

‘We had challenges because our students are older people who are not technologically savvy, so they had a problem with the online facilitation because some could not reach the necessary information from us lecturers due to financial constraints or technological illiteracy.’ (Participant D, Female, FGD 1)

Another said:

‘The other experience we had was the online teaching and learning regarding WhatsApp teaching and learning because most of the learners could not internalise the content, it was too difficult for them, and they became overwhelmed as also there were no group discussions for others to come and try to explain the content to each other.’ (Participant F, Female, FGD 1)

The statements indicate that the COVID-19 pandemic compelled HEIs to resort to technology to continue teaching and learning. However, technical challenges, such as poor digital skills and internet connectivity, needed improvement. Thus, this indicates that the NEI should be well established regarding IT resources.

### Theme 3: Strategies for overcoming the challenges experienced by nurse educators during the COVID-19 pandemic

Strategies for overcoming the challenges experienced by nurse educators during the COVID-19 pandemic were highlighted as the third theme. The following sub-themes emerged: compliance with all COVID-19 rules and regulations, vaccination of employees and students, and special arrangements for extra classes and remedial assessments.

#### Sub-theme 3.1: Compliance with all COVID-19 rules and regulations

Participants reported that to curb the spread of COVID-19, they had to abide by the rules and regulations. One participant said:

‘I think as we have alluded we complied with the rules and regulations of COVID-19 like decontamination at a frequent intervals and by screening ensuring that whoever comes into the institutions does not have a high temperature and does not cough and stuff like that.’ (Participant C, Female, FGD 1)

Another participant added:

‘Adding to it by complying uh, also by wearing of masks. Sometimes, it will be a problem with some of our students where they were not able to access uh, masks, the, the school was able to give some masks to the students when they are at the facilities thank you.’ (Participant E, Female, FGD 1)

This indicates that the NEI played a role in flattening the curve of COVID-19 infections by complying with the set rules and regulations.

#### Sub-theme 3.2: Vaccination of all employees and students

Participants reported that in order to curb the spread of COVID-19, the importance of vaccinations was emphasised among the employees of the institution as a whole.

One participant said:

‘To deal with our own personal fear of infections and also to reduce infection rate we aimed at reaching herd immunity. So, all staff members, educators, support staff, and even the students we encouraged to be immunised could not be forced, we were not coerced into doing that but there were presentations where they were encouraged and that was done and the, uh … uh …. educators, students, uh … staff members, were allowed to go to different clinical institutions where immunisation was done.’ (Participant A, Female, FGD 1)

One participant said:

‘So a lot of our staff members were immunised weekly, on Mondays there will be statistics and it was showing an increase in the number of people who were getting immunised.’ (Participant A, Female, FGD 1)

Another participant added:

‘I think the best way that can assist us is when all of us are vaccinated I mean all staff members and the students and everyone who is coming to the college must be vaccinated.’ (Participant E, Female, FGD 1)

The statements indicate that the NEI also supported the rule of herd immunity by encouraging the employees and students to vaccinate.

#### Sub-theme 3.3: Special arrangement for extra classes and remedial assessments

Participants reported that they had to devise measures to catch up with the students by conducting remedial classes.

One participant said:

‘Uh, with the catch up, you must also follow the regulations. You will have a contact session, but it must be in an open space which is in a classroom, and the student must maintain social distancing and so forth.’ (Participant B, Male, FGD 3)

Another participant added:

‘Some of them will be put off when we do a catch up until the exam. We will catch up when they are writing, maybe tomorrow or after tomorrow. You still have to catch up with some groups because of this COVID … And the remedial also, we got the catch-up, and we got the Remedial. The remedial for those who are supposed to write the optional test is the same as the catch-up.’ (Participant B, Female, FGD 4).

The statements indicate that irrespective of measures taken to continue teaching and learning during the COVID-19 pandemic, the problem of needing more contact sessions was experienced. Therefore, other measures had to be incorporated to catch up.

## Discussion

This study aimed to explore and describe the experiences of nurse educators regarding teaching and learning during the COVID-19 pandemic at a NEI in the NWP. The outbreak of COVID-19 was sudden leading to teaching and learning at NEIs in NWP being affected by rapid measures that were implemented to curb the spread of the disease. The lack of advanced technology skills at this NEI for teaching and learning compared to universities became apparent during the COVID-19 pandemic, and it brought about diverse lessons learned to prepare for future pandemics. Regardless of the challenges posed by COVID-19, the participants affirmed positive experiences regarding teaching and learning which were embraced by the NEI. Among the skills learned, there were improvements in digital competencies such as attending Zoom meetings, video conferencing and facilitating online classes via Zoom. These were innovative digital modes of teaching which the nurse educators were not conversant with before. These developments align with Sustainable Development Goal 4, which emphasises the importance of quality education that is inclusive, effective and equitable for all. The findings of this article are congruent with the findings of Kim, Kim and Lee (2021:11) and Makhado et al. (2022:6) who reported that participants were excited about the new mode of teaching and learning and new things learned every day.

The nurse educators reported that the COVID-19 pandemic generated new opportunities for utilising virtual education in nursing education (Tolyat, Vagharseyyedin & Nakhaei 2022:45). Prior to the COVID-19 pandemic, communication was only face to face. Therefore, the COVID-19 pandemic fostered an introduction of blended teaching and learning. This led the South African Nursing Council to later approve the NEI for both contact and remote learning. Additionally, the positive outcomes were not only affirmed by nurse educators as a study conducted by Oducado and Estoque (2021:148) indicated that more nursing students suggested combined online and contact teaching and learning methods.

Contrary to the positive outcomes of infusing technology in teaching and learning, nurse educators and nursing students experienced a plethora of challenges. Because of limited digital skills, teaching and learning could not continue during the COVID-19 pandemic at the NEI in the beginning during alert levels 4 and 5. This study revealed that nurse educators and nursing students were unfamiliar with online platforms like Zoom as the approved teaching mode was contact. Literature supports that the lack of online learning experience disadvantaged nursing students (Leaver et al. 2022:83). The study highlighted that the lack of use of technology in nursing education had a negative impact on learning outcomes, accessibility and preparedness. Therefore, infusing technology in teaching and learning could address such gaps, enhance the quality of nursing education and align with modern health care demands. Moreover, the use of technology may ensure continuity of teaching and learning during disruptions like COVID-19 in the future. Additionally, the digital divide underscores the socio-economic disparities within rural and low-income communities, where there is often a lack of access to technology, reliable internet and digital skills. These challenges are driven by inequalities related to geography, economics and insufficient infrastructure. The study revealed that some technical difficulties stemmed from the lack of resources, such as advanced mobile phones, which limited nursing student’s ability to access information on WhatsApp group chats that were used during the pandemic for communication with educators. Furthermore, poor internet connectivity was another significant challenge, particularly for students living in rural areas with limited network coverage. Similarly, Makhado et al. (2022:8) asserted that most nursing students come from rural areas without internet connectivity. Additionally, Molefe and Mabunda (2022:6) affirmed that nursing students of low socio-economic status could not afford to purchase sufficient data to connect; therefore, they experienced connectivity and class attendance difficulties.

The findings of this study indicated that the COVID-19 pandemic promoted teamwork among employees, intending to combat the deadly disease. Additionally, this study revealed that during the pandemic, people took roles that they would not do normally under usual circumstances. For example, security officers at the NEI in NWP were involved in screening and measuring body temperatures. The COVID-19 pandemic united people of different professions and categories in the face of a common challenge (Iheduru-Anderson & Foley 2021:7).

The nurse educators feared contracting COVID-19 and dying. The outbreak of the COVID-19 pandemic claimed many people’s lives worldwide, which led to many people being fearful for their lives as well as passing the disease to their families. The clinical accompaniment of students by nurse educators aggravated the fear of contracting COVID-19 and dying. Because of the measures that were implemented to reduce the spread of the disease such as reduction of overcrowding, the nursing students were placed in small numbers for clinical services, which forced the nurse educators to visit clinical facilities more often than usual. This, however, increased their risk of contracting the disease. Similarly, other studies revealed that nurse educators and nursing students experienced worries and uncertainties about contracting the COVID-19 disease or transmitting it to their families (Farsi et al. 2021:7; Kaveh et al. 2022:6).

The findings also revealed the necessity for additional classrooms. One of the COVID-19 rules and regulations was that indoor venues should be filled only to 50% of their capacity. The NEI had to outsource venues to continue teaching and learning. However, these venues were not always conducive to teaching and learning, as some lacked essential infrastructure. For example, there was no backup electricity or internet connectivity. Consistent with the findings of this study, a study by Peneza and colleagues (2021:184) reported that the infrastructure in some venues could not accommodate the required social distancing of 1.5 m to 2 m. Moreover, students had to be divided into groups, resulting in the exhaustion of nurse educators as a result of repeating the same content to different groups. Makhado et al. (2022:5) alluded that repeating classes consumes much time and is strenuous for the presenter. It further increases the chances of inconsistency and missing some information for other groups.

The abrupt change in theoretical and clinical teaching and learning methods had an increased detriment to teaching and learning. The NEI was compelled to restructure the assessment tools that were prepared before the pandemic to accommodate the rules and regulations of COVID-19. Similarly, in other areas, the NEIs were forced to amend the teaching and learning of clinical skills, including restructuring clinical setup and assessment approaches because of the pandemic (Lobão et al. 2023:378; Tolyat et al. 2022:45). These changes affected the length of the academic year which resulted in the extension of all nursing programmes. This extension was a significant challenge to the NEI where students’ training was prolonged even though they did not fail. These findings are consistent with a study conducted by Michel et al. (2021:904), who reported that restricting students’ access to clinical sites delayed their completion of nursing programmes within the expected time frame.

Furthermore, because of the challenges of inadequate IT infrastructure, the selected NEI experienced problems with the continuation of both theoretical and clinical teaching and learning, irrespective of the measures implemented (Oducado & Estoque 2021:149). Moreover, there were delays in a clinical context, as during the different COVID-19 lockdowns, students were not permitted at clinical facilities depending on the level of lockdown. This affected the expected clinical contact hours required by SANC. The clinical component is vital in training nursing students whose teaching and learning include the correlation of theory and practice (Kim et al. 2021:2). Therefore, NEIs are expected to ensure that nursing students complete the required clinical learning outcomes and hours.

The selected NEI abided by the rules and regulations of COVID-19 that were instituted to curb the spread of COVID-19, which comprises sanitising of hands, wearing of masks, screening and decontamination of surfaces. Most countries worldwide emphasised health practices such as wearing masks, social distancing and restrictions on travelling and mass gatherings (Rab et al. 2020:1617; Tolyat et al. 2022:45). Complying with health practices was proven to reduce the spread of COVID-19 disease. One of the ways to curb the spread of COVID-19 disease was vaccination. This study’s findings revealed that the nurse educators, nursing students and other staff members of the selected NEI were encouraged to vaccinate against COVID-19. Nonetheless, it was not compulsory. Coronavirus disease 2019 vaccination was regarded as vital for the population’s health and safety (Cooper, Van Rooyen & Wiysonge 2021:921). Vaccination in communities reduced the rate of COVID-19 infections and deaths. Moreover, there was a reduction in health care costs because of fewer hospitalisations (Reddy et al. 2021:1). Encouraging vaccinations was to accomplish herd immunity which offers unvaccinated individuals some protection, thus curbing the spread of COVID-19 infections (Dzinamarira et al. 2021:3).

Additionally, the herd community supported the country’s aim of a minimum of 67% population vaccinations. This study revealed that to catch up for the work lost during COVID-19 lockdowns, measures such as arrangements of special classes had to be initiated. Moreover, remedial classes were also conducted before writing theoretical assessments and even for the students writing second-opportunity assessments. Extension of programmes was unavoidable as the measures incorporated to continue teaching and learning were unsuccessful because of technical challenges (Molefe & Mabunda 2022:7).

### Limitation

This study was conducted at one selected NEI with two campuses, and it could yield different results when conducted at well-established institutions such as universities or at any of the other public and private NEIs. Therefore, this study cannot be representative of all the NEIs; it could yield different findings when conducted in different provinces.

## Conclusion

The COVID-19 pandemic disrupted global educational activities and compelled nurse educators to adopt contemporary and evolving technologies to sustain teaching and learning during the pandemic when contact sessions were not permitted. Poor IT infrastructure and digital skills disrupted teaching and learning at the selected NEI. There is a need for nurse educators and nursing students to be conversant with innovative digital teaching and learning skills. Therefore, adding a basic computer literacy module to the curriculum could be considered to continue teaching and learning as a measure to prepare for prospective pandemics. Furthermore, policymakers may use the findings to formulate HEI policies for teaching and learning as future guidelines for any prospective pandemics.
